# Quality of Life Among Women With Breast Cancer

**DOI:** 10.1192/j.eurpsy.2022.2209

**Published:** 2022-09-01

**Authors:** A. Chouchane, I. Kacem, I. Bannour, M. Kahloul, M. Maoua, W. Naija, N. Mrizak

**Affiliations:** 1 Farhat Hached Academic Hospital, Occupational Medicine, Sousse, Tunisia; 2 Farhat Hached Academic Hospital, Gynecology And Obstetrics, Sousse, Tunisia; 3 Sahloul Academic Hospital, University of medicine, “Ibn Al Jazzar”, Sousse, Tunisia, Department Of Anesthesia And Intensive Care,, Sousse, Tunisia

**Keywords:** Quality of Life, breast cancer, women

## Abstract

**Introduction:**

Breast cancer (BC) is the most common cancer in women all over the world. Its physical consequences and psychosocial distress had adverse effect on quality of life (QOL).

**Objectives:**

Evaluate the QOL among women with BC.

**Methods:**

Descriptive cross-sectional study of BC patients, carried out during a period of 5 years. Socio-demographic and medical data was collected based on a pre-established synoptic sheet. The European Organization for Research and Treatment Quality of Life Questionnaires (EORTC QLQ-C30) and EORTC QLQ-BR23 were used to assess the QOL.

**Results:**

A total of 100 patients were included. The mean age was 52 ± 8 years. The mean overall health score was 77.5 ± 25.5. The mean scores of physical and emotional functioning were 82 ± 25.1 and 90 ± 19.5 respectively. The QLQ-BR23 objectified a body image score of 63 ± 23.9, an average score for sexual functioning of 87.3 ± 22 with loss of sexual enjoyment in 75% of cases. The increase in mean overall health score and physical functioning was significantly associated with type of treatment (p = 0.01). The mean score of emotional functioning was correlated with the feeling of support (p = 0.04). The increase in the patient’s body image score was significantly associated with age (p <10-3), marital status (p = 0.01) and having had a mastectomy (p <10-3).

**Conclusions:**

Our results underline the importance of psychosocial care, which must be an integral part of oncological care in women with BC, in order to improve their quality of life.

**Disclosure:**

No significant relationships.

